# Supplementary material for: Accelerating Leigh syndrome drug discovery through deep learning screening in brain organoids
Source: Nat Commun. 2026 Apr 20;17:3570. doi: 10.1038/s41467-026-71391-2 (PMC13096141; doi:10.1038/s41467-026-71391-2)
Supplement: Supplementary file 1 — Supplementary Information [file 41467_2026_71391_MOESM1_ESM.pdf]

SUPPLEMENTARY INFORMATION

Accelerating Leigh syndrome drug discovery through deep learning screening in brain organoids

Menacho, Okawa et al.

Manuscript correspondence:

Alessandro Prigione, alessandro.prigione@hhu.de

Antonio del Sol, antonio.delsol@uni.lu

Description of Supplementary Information

Supplementary Figure 1. Development of deep learning (DL) algorithm for screening of compounds (related to Figure 1).

Supplementary Figure 2. Development of yeast viability assay and neuromorphogenesis validation in Leigh models with DL-predicted drugs (DLDs) and yeast screen drugs (YSDs) (related to Figure 2).

Supplementary Figure 3. Characterization of midbrain organoids (MOs) generated in this study (related to Figure 3).

Supplementary Figure 4. Single-cell RNA sequencing (scRNAseq) of MOs (related to Figure 3).

Supplementary Figure 5. Altered biological processes and calcium imaging in Leigh MOs (related to Figure 3).

Supplementary Figure 6. Effects of sertaconazole and talarozole in Leigh MOs (related to Figure 4).

Supplementary Figure 7. Sertaconazole and talarozole modulate lipid metabolism in Leigh neural models (related to Figure 4 and Figure 5).

Supplementary Figure 8. Effect of sertaconazole and talarozole on energy metabolism in Leigh NPCs (related to Figure 5).

Supplementary Figure 9. Talarozole and sertaconazole outperform other azoles in binding CYP120 pocket (related to Figure 6).

Supplementary Figure 10. SMINA docking affinity scores of talarozole and sertaconazole to CYP26 and PPARγ compared to other azole compounds (related to Figure 6).

Supplementary Figure 11. Original blots for SNPH, KIF5A and β-Actin (related to Supplementary Figure 6).

Supplementary Figure 12. Overview of the study.

Supplementary Table 1. Small molecule screening data in yeast

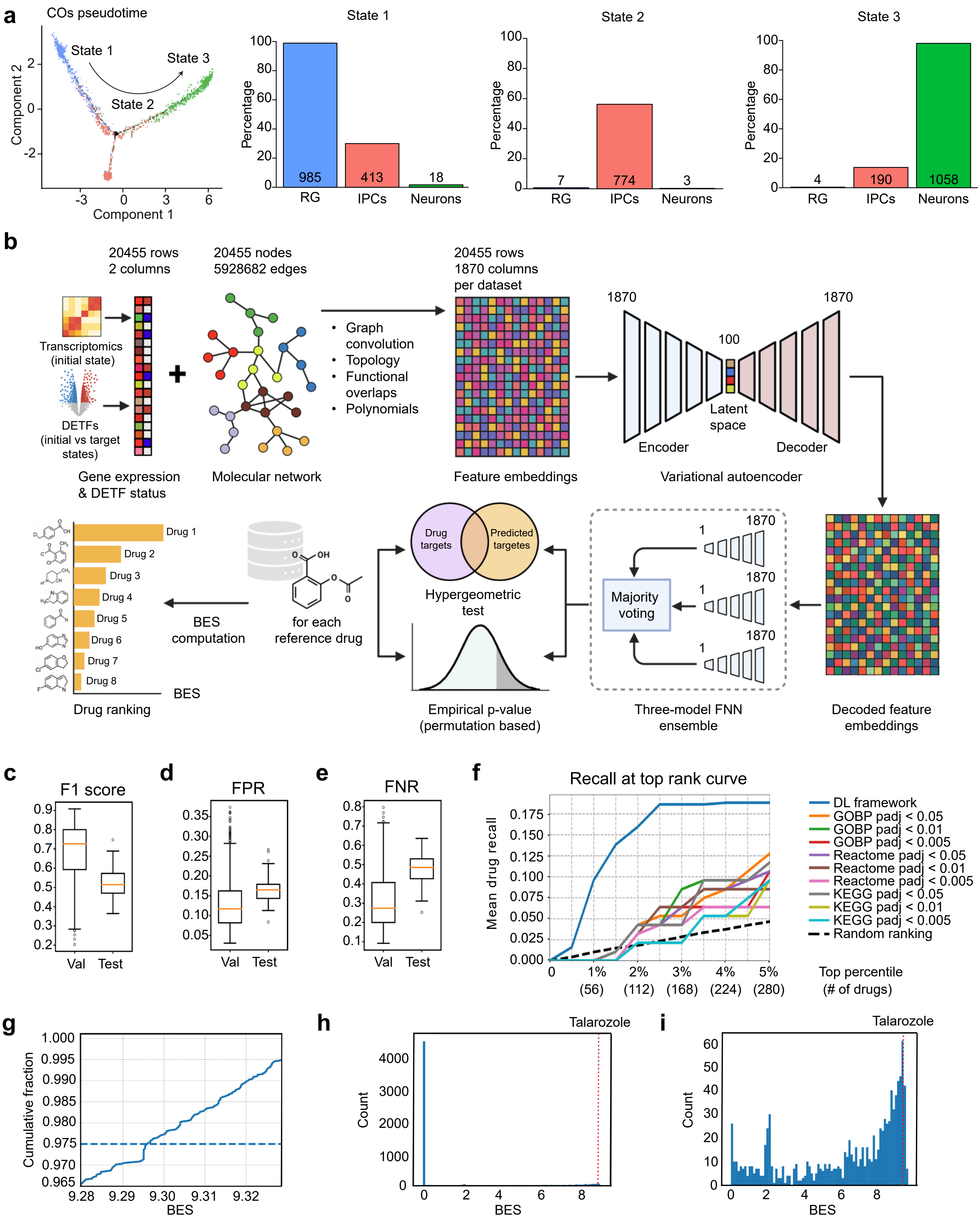
a
COs pseudotime
State 1
State 2
State 3
Component 1
Component 2
Percentage
RG
IPCs
Neurons
985
413
18
7
774
3
4
190
1058
b
20455 rows
2 columns
20455 nodes
5928682 edges
20455 rows
1870 columns
per dataset
Transcriptomics
(initial state)
DETFs
(initial vs target
states)
Gene expression
& DETF status
Molecular network
Graph convolution
Topology
Functional overlaps
Polynomials
Feature embeddings
1870
100
1870
Latent space
Encoder
Decoder
Variational autoencoder
Drug targets
Predicted targetes
Hypergeometric test
Majority voting
Three-model FNN ensemble
Decoded feature embeddings
Empirical p-value
(permutation based)
for each
reference drug
BES computation
Drug 1
Drug 2
Drug 3
Drug 4
Drug 5
Drug 6
Drug 7
Drug 8
BES
Drug ranking
c
F1 score
d
FPR
e
FNR
Val
Test
f
Recall at top rank curve
Mean drug recall
DL framework
GOBP padj < 0.05
GOBP padj < 0.01
GOBP padj < 0.005
Reactome padj < 0.05
Reactome padj < 0.01
Reactome padj < 0.005
KEGG padj < 0.05
KEGG padj < 0.01
KEGG padj < 0.005
Random ranking
Top percentile
(# of drugs)
0
1% (56)
2% (112)
3% (168)
4% (224)
5% (280)
g
Cumulative fraction
BES
h
Talarozole
Count
BES
i
Talarozole
Count
BES

Supplementary Figure 1. Development of deep learning (DL) algorithm for screening of compounds (related to Figure 1). (a) Percent distribution of radial glia (RG) (cluster 7), intermediate progenitor cells (IPCs) (cluster 4) and neurons (cluster 6) at different pseudotime states in Leigh COs and isogenic control COs. **(b)** Schematic overview of the DL-based drug repurposing framework deployment. The framework integrates differentially expressed transcription factors (DETFs) between initial and target states and leverages graph-based embeddings to infer protein-level perturbations and drugs that shift the system toward the desired transcriptional target state. BES: Bayesian enrichment score (created in BioRender. Okawa, S. (2026) https://BioRender.com/nj5lju7). **(c-e)** Classification performance of the ensembled feedforward neural network (FNN) model on the validation (10 % hold-out) and independent test datasets. Performance metrics include F1 score, false positive rate (FPR), and false negative rate (FNR). Orange lines indicate the median. **(f)** Mean drug recall at top-ranked predictions, comparing the DL-based framework against conventional gene set enrichment analysis (GSEA)-based methods using Gene Ontology Biological Processes (GOBP), Reactome, and KEGG pathway annotations at varying significance thresholds. Black dotted line indicates randomized ranking baseline. **(g)** Empirical cumulative distribution function (ECDF) of BES illustrating the distribution of DL framework-based drug prioritization values with the high-score tail showing a consistent inflection ("knee") at approximately the 97.5th percentile. The dashed lines indicate the 97.5th percentile threshold, which was used to define the top 2.5 % high-confidence candidates. **(h)** Histogram of BES for all 5,692 screened compounds in the DETF1 comparison. **(i)** Histogram of BES in the DETF1 comparison restricted to the non-zero subset. The red dashed vertical line marks the BES of talarozole (BES = 9.034277).

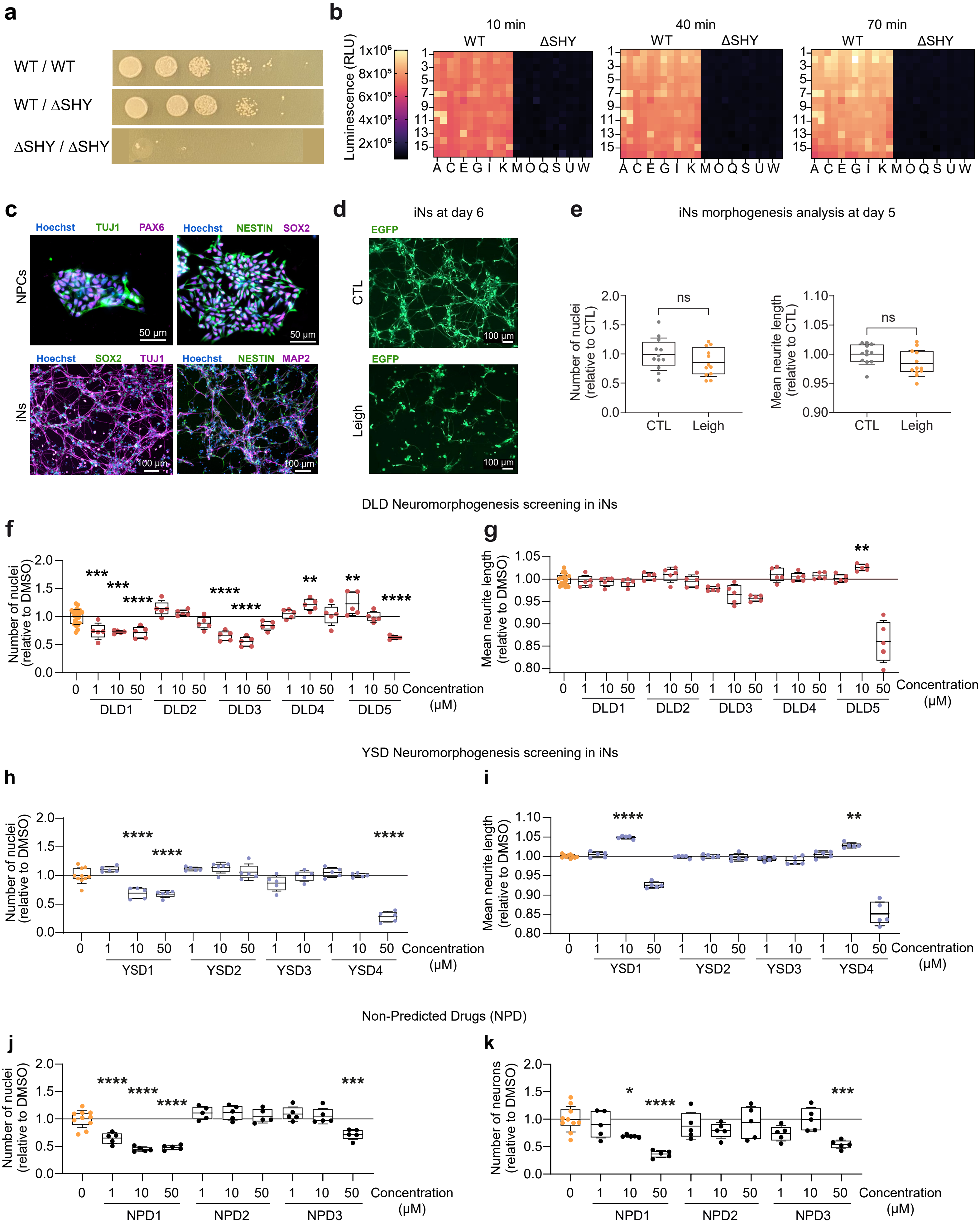
a
WT / WT
WT / ΔSHY
ΔSHY / ΔSHY
b
10 min
40 min
70 min
WT
ΔSHY
Luminescence (RLU)
1x10⁶
8x10⁵
6x10⁵
4x10⁵
2x10⁵
A C E G I K M O Q S U W
c
Hoechst TUJ1 PAX6
Hoechst NESTIN SOX2
Hoechst SOX2 TUJ1
Hoechst NESTIN MAP2
NPCs
iNs
50 μm
100 μm
d
iNs at day 6
EGFP
CTL
Leigh
100 μm
e
iNs morphogenesis analysis at day 5
Number of nuclei (relative to CTL)
Mean neurite length (relative to CTL)
ns
CTL
Leigh
DLD Neuromorphogenesis screening in iNs
f
Number of nuclei (relative to DMSO)
g
Mean neurite length (relative to DMSO)
DLD1
DLD2
DLD3
DLD4
DLD5
Concentration (μM)
YSD Neuromorphogenesis screening in iNs
h
Number of nuclei (relative to DMSO)
i
Mean neurite length (relative to DMSO)
YSD1
YSD2
YSD3
YSD4
Concentration (μM)
Non-Predicted Drugs (NPD)
j
Number of nuclei (relative to DMSO)
k
Number of neurons (relative to DMSO)
NPD1
NPD2
NPD3
Concentration (μM)

Supplementary Figure 2. Development of yeast viability assay and neuromorphogenesis validation in Leigh models with DL-predicted drugs (DLDs) and yeast screen drugs (YSDs) (related to Figure 2). (a-b) Viability assay in cultures from wild-type (WT) yeast and from yeast knock-out for the *SURF1* homologue *SHY1* (ΔSHY). **(c)** Representative immunostainings of control NPCs (top) expressing early neural progenitor markers (SOX2, NESTIN and PAX6) and iNs after 7 days of induction expressing neuronal markers TUJ1 and MAP2 (bottom). **(d)** Neurite outgrowth defects in Leigh iNs after NGN2 induction based on EGFP signal that is part of the NGN2 inducible cassette. Scale bars: 100 µm. **(e)** Quantification of number of nuclei (left) and mean neurite length (right) in Leigh iNs related to isogenic control (CTL) iNs using high-content analysis (HCA) of nuclear (Hoechst) and dendritic (MAP2) markers, respectively. Boxplots: mean (center), 25/75 percentiles (box), and 1x SD (whiskers). Individual dots: n=12 biological replicates; *p<0.05, unpaired two-tailed t test. **(f-i)** Neuromorphogenesis screen for DLDs (f, g) and YSDs (h, i) in Leigh iNs based on HCA quantification of number of nuclei and mean neurite length related to DMSO-treated iNs ("0"). Boxplots: mean (center), 25/75 percentiles (box), and 1x SD (whiskers). Individual dots: n=5 biological replicates, related to DMSO-treated conditions; **p<0.01, ***p<0.005, ****p<0.001, one-way ANOVA, compound-treated Leigh iNs vs DMSO-treated Leigh iNs. **(j-k)** Number of Leigh iNs treated with DMSO or non-predicted drugs (NPDs) from the DL screen. Boxplots: mean (center), 25/75 percentiles (box), and 1x SD (whiskers). Individual dots: n=5 biological replicates, related to DMSO-treated conditions; ns: not significant, one-way ANOVA, NPD-treated Leigh iNs vs DMSO-treated Leigh iNs. Source data are provided as a Source Data file.

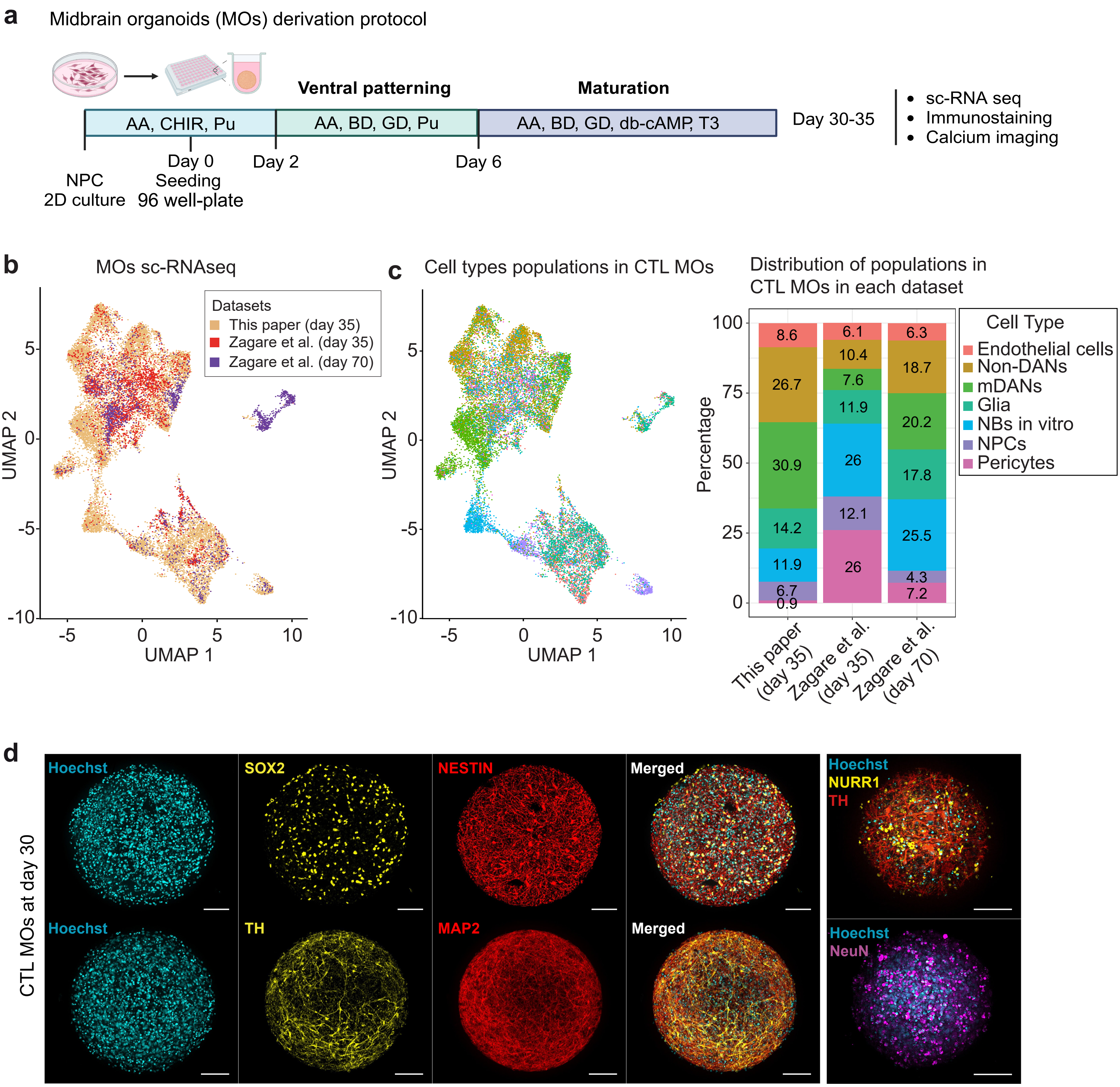
a
Midbrain organoids (MOs) derivation protocol
Ventral patterning
Maturation
AA, CHIR, Pu
AA, BD, GD, Pu
AA, BD, GD, db-cAMP, T3
Day 30-35
sc-RNA seq
Immunostaining
Calcium imaging
Day 0
Day 2
Day 6
NPC
2D culture
Seeding
96 well-plate
b
MOs sc-RNAseq
Datasets
This paper (day 35)
Zagare et al. (day 35)
Zagare et al. (day 70)
UMAP 1
UMAP 2
c
Cell types populations in CTL MOs
Distribution of populations in CTL MOs in each dataset
Percentage
Cell Type
Endothelial cells
Non-DANs
mDANs
Glia
NBs in vitro
NPCs
Pericytes
This paper (day 35)
Zagare et al. (day 35)
Zagare et al. (day 70)
8.6
26.7
30.9
14.2
11.9
6.7
0.9
6.1
10.4
7.6
11.9
26
12.1
26
6.3
18.7
20.2
17.8
25.5
4.3
7.2
d
CTL MOs at day 30
Hoechst
SOX2
NESTIN
Merged
Hoechst
TH
MAP2
Merged
Hoechst
NURR1
TH
Hoechst
NeuN

Supplementary Figure 3. Characterization of midbrain organoids (MOs) generated in this study (related to Figure 3). (a) Schematic of MOs protocol derived from NPCs (created in BioRender. Menacho, C. (2026) https://BioRender.com/8kuusx7). **(b-c)** Unsupervised clustering and population distribution of scRNAseq of our MOs ("This paper") compared to previously derived MOs ("Zagare et al") at different time points (day 35 and day 70) showing high proportion (30.9 %) of midbrain dopaminergic neurons (mDANs) markers in MOs used in this study at day 35 compared to Zagare et al. at day 35 and 70 (7.6 % and 20.2 %, respectively). **(d)** Immonostaining images of day 30 CTL MOs expressing neural progenitor markers (SOX2, NESTIN), neuronal markers (NeuN, MAP2), and dopaminergic neuron markers (TH, NURR1). Scale bars: 100 μm.

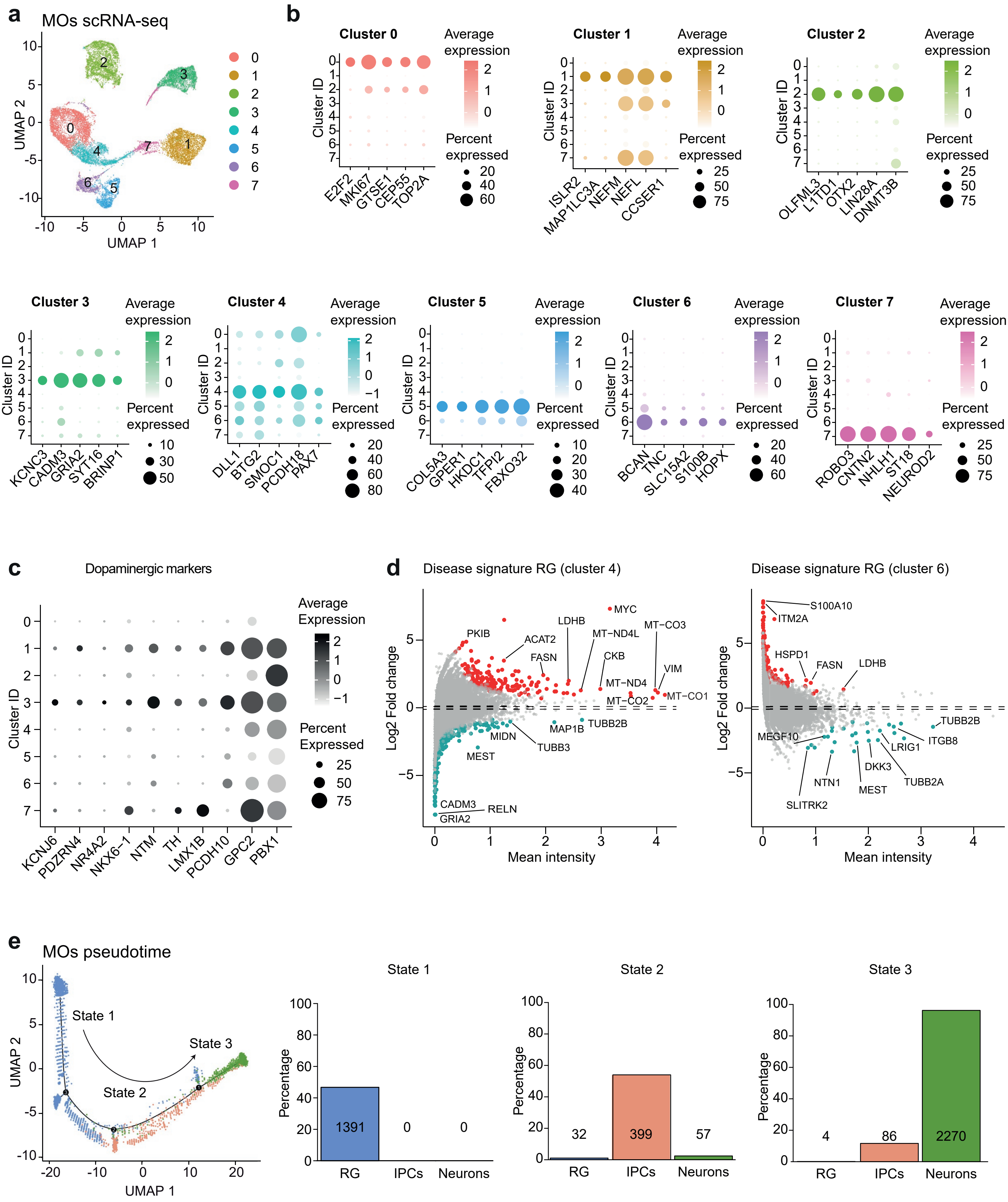

a
MOs scRNA-seq
UMAP 1
UMAP 2
b
Cluster 0
Cluster 1
Cluster 2
Cluster 3
Cluster 4
Cluster 5
Cluster 6
Cluster 7
Cluster ID
Average expression
Percent expressed
E2F2 MKI67 GTSE1 CEP55 TOP2A
ISLR2 MAP1LC3A NEFM NEFL CCSER1
OLFML3 L1TD1 OTX2 LIN28A DNMT3B
KCNC3 CADM3 GRIA2 SYT16 BRINP1
DLL1 BTG2 SMOC1 PCDH18 PAX7
COL5A3 GPER1 HKDC1 TFPI2 FBXO32
BCAN TNC SLC15A2 S100B HOPX
ROBO3 CNTN2 NHLH1 ST18 NEUROD2
c
Dopaminergic markers
Average Expression
Percent Expressed
Cluster ID
KCNJ6 PDZRN4 NR4A2 NKX6-1 NTM TH LMX1B PCDH10 GPC2 PBX1
d
Disease signature RG (cluster 4)
Disease signature RG (cluster 6)
Log2 Fold change
Mean intensity
MYC PKIB LDHB ACAT2 MT-ND4L MT-CO3 FASN CKB VIM MT-ND4 MT-CO2 MT-CO1 MAP1B TUBB2B MIDN TUBB3 MEST CADM3 RELN GRIA2
S100A10 ITM2A HSPD1 FASN LDHB TUBB2B MEGF10 ITGB8 LRIG1 DKK3 TUBB2A NTN1 MEST SLITRK2
e
MOs pseudotime
State 1
State 2
State 3
UMAP 1
UMAP 2
Percentage
RG IPCs Neurons
1391 0 0
32 399 57
4 86 2270

Supplementary Figure 4. Single-cell RNA sequencing (scRNAseq) of MOs (related to Figure 3). (a) Unsupervised clustering of CTL MOs and Leigh MOs revealing 8 clusters. **(b)** Dot plot featuring five representative genes for each of the 8 clusters present within MOs. **(c)** Dot plot depicting the expression of dopaminergic markers within each identified cluster, predominantly expressed in clusters 1, 3 and 7, corresponding to Leigh neurons, CTL neurons and IPCs, respectively. **(d)** Volcano plot of pseudo-bulk analysis of scRNAseq dataset depicting the disease signature in the radial glia (RG) population (clusters 4 and 6). **(e)** Percent distribution of RG (clusters 4-6), intermediate progenitor cells (IPCs) (cluster 7) and neurons (cluster 3) at different pseudotime states in Leigh MOs and CTL MOs.

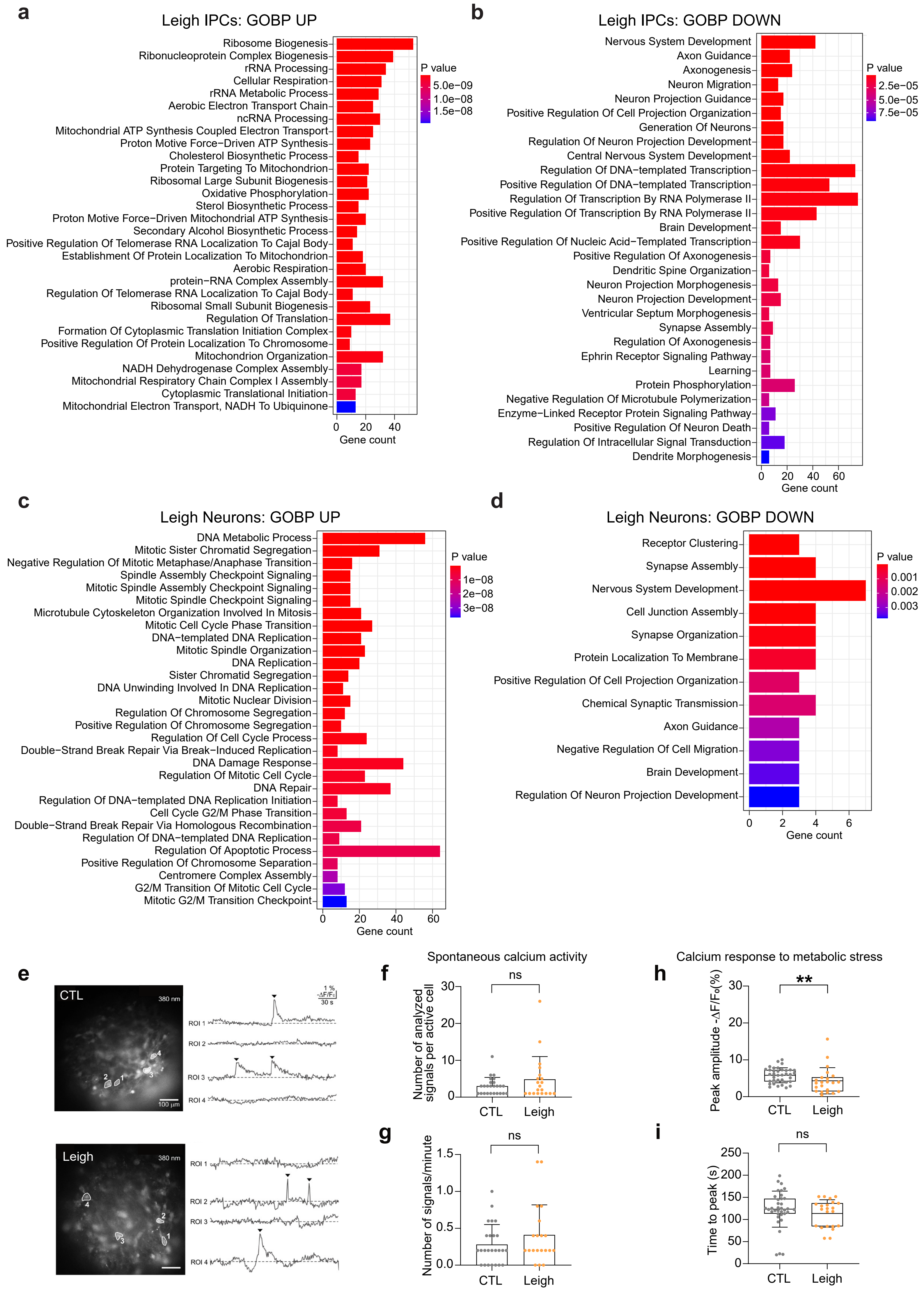
a
Leigh IPCs: GOBP UP
Ribosome Biogenesis
Ribonucleoprotein Complex Biogenesis
rRNA Processing
Cellular Respiration
rRNA Metabolic Process
Aerobic Electron Transport Chain
ncRNA Processing
Mitochondrial ATP Synthesis Coupled Electron Transport
Proton Motive Force−Driven ATP Synthesis
Cholesterol Biosynthetic Process
Protein Targeting To Mitochondrion
Ribosomal Large Subunit Biogenesis
Oxidative Phosphorylation
Sterol Biosynthetic Process
Proton Motive Force−Driven Mitochondrial ATP Synthesis
Secondary Alcohol Biosynthetic Process
Positive Regulation Of Telomerase RNA Localization To Cajal Body
Establishment Of Protein Localization To Mitochondrion
Aerobic Respiration
protein−RNA Complex Assembly
Regulation Of Telomerase RNA Localization To Cajal Body
Ribosomal Small Subunit Biogenesis
Regulation Of Translation
Formation Of Cytoplasmic Translation Initiation Complex
Positive Regulation Of Protein Localization To Chromosome
Mitochondrion Organization
NADH Dehydrogenase Complex Assembly
Mitochondrial Respiratory Chain Complex I Assembly
Cytoplasmic Translational Initiation
Mitochondrial Electron Transport, NADH To Ubiquinone
0
20
40
Gene count
P value
5.0e−09
1.0e−08
1.5e−08
b
Leigh IPCs: GOBP DOWN
Nervous System Development
Axon Guidance
Axonogenesis
Neuron Migration
Neuron Projection Guidance
Positive Regulation Of Cell Projection Organization
Generation Of Neurons
Regulation Of Neuron Projection Development
Central Nervous System Development
Regulation Of DNA−templated Transcription
Positive Regulation Of DNA−templated Transcription
Regulation Of Transcription By RNA Polymerase II
Positive Regulation Of Transcription By RNA Polymerase II
Brain Development
Positive Regulation Of Nucleic Acid−Templated Transcription
Positive Regulation Of Axonogenesis
Dendritic Spine Organization
Neuron Projection Morphogenesis
Neuron Projection Development
Ventricular Septum Morphogenesis
Synapse Assembly
Regulation Of Axonogenesis
Ephrin Receptor Signaling Pathway
Learning
Protein Phosphorylation
Negative Regulation Of Microtubule Polymerization
Enzyme−Linked Receptor Protein Signaling Pathway
Positive Regulation Of Neuron Death
Regulation Of Intracellular Signal Transduction
Dendrite Morphogenesis
0
20
40
60
Gene count
P value
2.5e−05
5.0e−05
7.5e−05
c
Leigh Neurons: GOBP UP
DNA Metabolic Process
Mitotic Sister Chromatid Segregation
Negative Regulation Of Mitotic Metaphase/Anaphase Transition
Spindle Assembly Checkpoint Signaling
Mitotic Spindle Assembly Checkpoint Signaling
Mitotic Spindle Checkpoint Signaling
Microtubule Cytoskeleton Organization Involved In Mitosis
Mitotic Cell Cycle Phase Transition
DNA−templated DNA Replication
Mitotic Spindle Organization
DNA Replication
Sister Chromatid Segregation
DNA Unwinding Involved In DNA Replication
Mitotic Nuclear Division
Regulation Of Chromosome Segregation
Positive Regulation Of Chromosome Segregation
Regulation Of Cell Cycle Process
Double−Strand Break Repair Via Break−Induced Replication
DNA Damage Response
Regulation Of Mitotic Cell Cycle
DNA Repair
Regulation Of DNA−templated DNA Replication Initiation
Cell Cycle G2/M Phase Transition
Double−Strand Break Repair Via Homologous Recombination
Regulation Of DNA−templated DNA Replication
Regulation Of Apoptotic Process
Positive Regulation Of Chromosome Separation
Centromere Complex Assembly
G2/M Transition Of Mitotic Cell Cycle
Mitotic G2/M Transition Checkpoint
0
20
40
60
Gene count
P value
1e−08
2e−08
3e−08
d
Leigh Neurons: GOBP DOWN
Receptor Clustering
Synapse Assembly
Nervous System Development
Cell Junction Assembly
Synapse Organization
Protein Localization To Membrane
Positive Regulation Of Cell Projection Organization
Chemical Synaptic Transmission
Axon Guidance
Negative Regulation Of Cell Migration
Brain Development
Regulation Of Neuron Projection Development
0
2
4
6
Gene count
P value
0.001
0.002
0.003
e
CTL
380 nm
100 µm
ROI 1
ROI 2
ROI 3
ROI 4
1 %
-ΔF/F₀
30 s
Leigh
380 nm
ROI 1
ROI 2
ROI 3
ROI 4
f
Spontaneous calcium activity
ns
Number of analyzed signals per active cell
0
10
20
30
CTL
Leigh
g
ns
Number of signals/minute
0.0
0.5
1.0
1.5
CTL
Leigh
h
Calcium response to metabolic stress
**
Peak amplitude -ΔF/F₀(%)
0
10
20
30
CTL
Leigh
i
ns
Time to peak (s)
0
50
100
150
200
250
CTL
Leigh

Supplementary Figure 5. Altered biological processes and calcium imaging in Leigh MOs (related to Figure 3). (a-b) Gene Ontology (GO) biological processes upregulated and downregulated in Leigh IPCs compared to CTL IPCs within MOs. **(c-d)** GO biological processes upregulated and downregulated in Leigh neurons compared to CTL neurons within MOs. **(e)** Widefield image of Fura2-loaded cells in CTL MOs (top) and Leigh MOs (bottom) with delineated regions of interest (ROIs) displaying spontaneous calcium signals during a 4 min measurement (1 Hz). Scale bar: 100 μm. **(f-g)** Spontaneous calcium activity in CTL MOs and Leigh MOs. Bar plots: mean +/- SD. Dots: individual cells within MOs (CTL: 41 cells from 7 MOs, Leigh: 35 cells from 6 MOs); n=3 independent experiments; $^{**}p<0.01$, $^{***}p<0.005$, $^{****}p<0.001$, Mann-Whitney U test. **(h-i)** Peak amplitude (top) and time to peak (bottom) of the metabolic stress-induced calcium signals. Boxplots: mean (center), 25/75 percentiles (box), and 1x SD (whiskers). Dots: individual cells within MOs (CTL: 36 cells from 7 MOs, Leigh: 22 cells from 6 MOs). Source data are provided as a Source Data file.

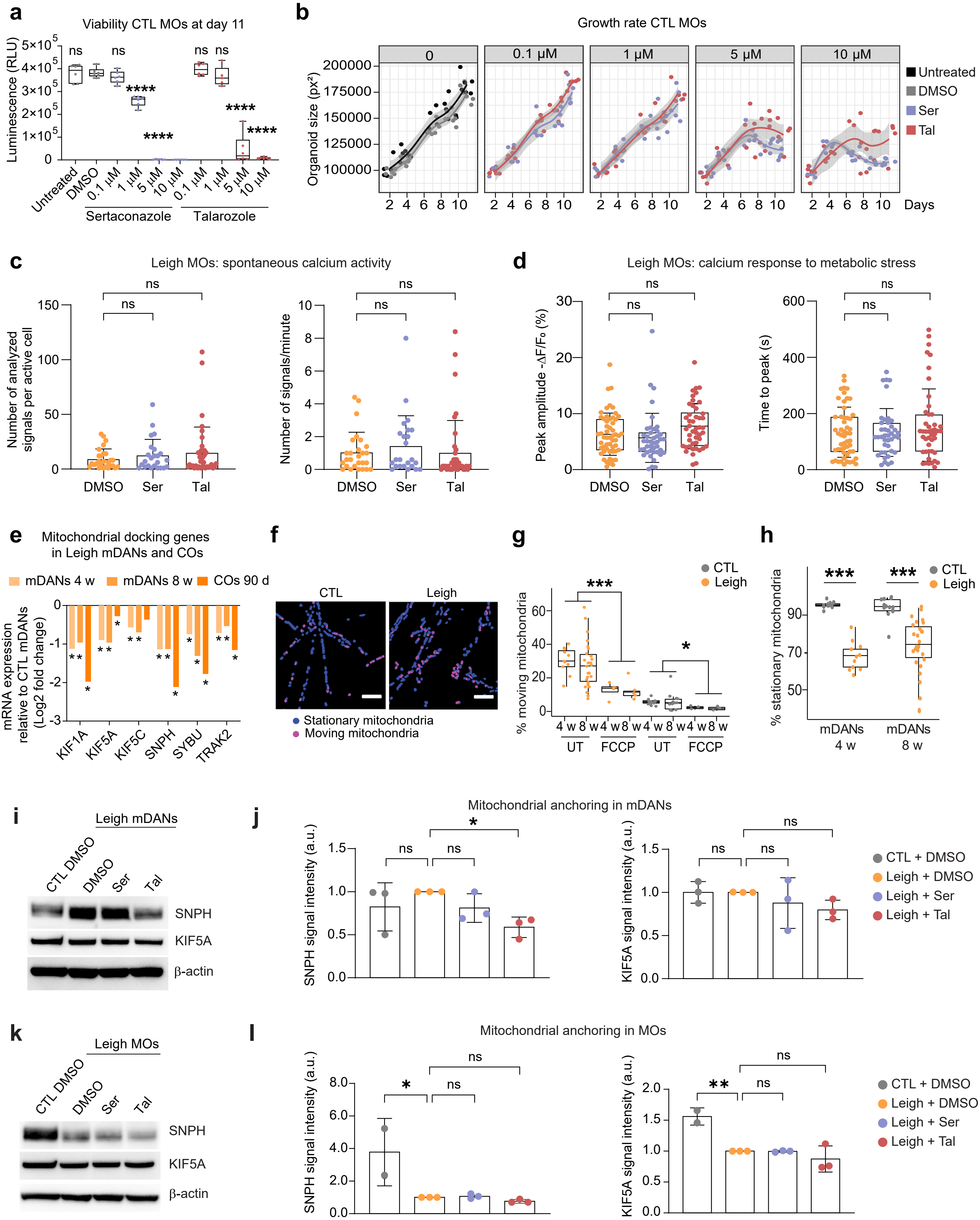

a
Viability CTL MOs at day 11
Luminescence (RLU)
Untreated
DMSO
0.1 µM
1 µM
5 µM
10 µM
Sertaconazole
Talarozole
ns
****
b
Growth rate CTL MOs
Organoid size (px²)
0
0.1 µM
1 µM
5 µM
10 µM
Days
Untreated
DMSO
Ser
Tal
c
Leigh MOs: spontaneous calcium activity
Number of analyzed signals per active cell
Number of signals/minute
DMSO
Ser
Tal
ns
d
Leigh MOs: calcium response to metabolic stress
Peak amplitude -ΔF/F₀ (%)
Time to peak (s)
e
Mitochondrial docking genes in Leigh mDANs and COs
mDANs 4 w
mDANs 8 w
COs 90 d
mRNA expression relative to CTL mDANs (Log2 fold change)
KIF1A
KIF5A
KIF5C
SNPH
SYBU
TRAK2
f
CTL
Leigh
Stationary mitochondria
Moving mitochondria
g
% moving mitochondria
CTL
Leigh
4 w
8 w
UT
FCCP
h
% stationary mitochondria
mDANs 4 w
mDANs 8 w
i
Leigh mDANs
CTL DMSO
DMSO
Ser
Tal
SNPH
KIF5A
β-actin
j
Mitochondrial anchoring in mDANs
SNPH signal intensity (a.u.)
KIF5A signal intensity (a.u.)
CTL + DMSO
Leigh + DMSO
Leigh + Ser
Leigh + Tal
k
Leigh MOs
l
Mitochondrial anchoring in MOs

Supplementary Figure 6. Effects of sertaconazole and talarozole in Leigh MOs (related to Figure 4). (a-b) Viability of control MOs showing that 0.1 μM sertaconazole and 1 μM talarozole as the highest non-toxic concentrations. Box plots: median (center line), interquartile range (box, 25th–75th percentiles), and minimum to maximum values (whiskers). Dots: individual biological replicates (n=5 control MOs); ****p<0.001, unpaired two-tailed *t* test **(c)** Spontaneous calcium activity in Leigh MOs treated with DMSO, sertaconazole (Ser) or talarozole (Tal). Bar plots: mean +/- SD. Dots: individual cells within MOs (DMSO: 56 cells from 9 MOs; Ser: 50 cells from 8 MOs; Tal: 48 cells from 8 MOs); n=3 independent experiments; **p<0.01, ***p<0.005, ****p<0.001, Mann-Whitney U test. **(d)** Peak amplitude (left) and time to peak (right) of the metabolic stress-induced calcium signals in treated Leigh MOs. Boxplots: mean (center), 25/75 percentiles (box), and 1x SD (whiskers). Dots: individual cells within MOs (DMSO: 54 cells from 9 MOs; Ser: 42 cells from 7 MOs; Tal: 46 cells infrom 7 MOs); n=3 independent experiments. **(e)** Expression of anchoring genes in Leigh mDANs and Leigh COs compared to isogenic controls. *p<0.01, fold change expression from bulk RNAseq dataset. **(f-h)** Mitochondrial motility in Leigh mDANs and CTL mDANs at 4 weeks (4w) or 8 weeks (8w) of differentiation starting from NPCs. Dots: individual mDANs; n=3 independent experiments; ***p<0.005; unpaired two-tailed t test, Leigh mDANs vs control mDANs. Scale bars: 100 μm. **(i-l)** Representative immunoblot and related quantification of mitochondrial anchoring proteins SNPH and KIF5A in treated mDANS and MOs. Bar plots: mean +/- SD of the signal intensity relative to Leigh + DMSO and normalized to β-actin (dots, n=3). Source data are provided as a Source Data file.

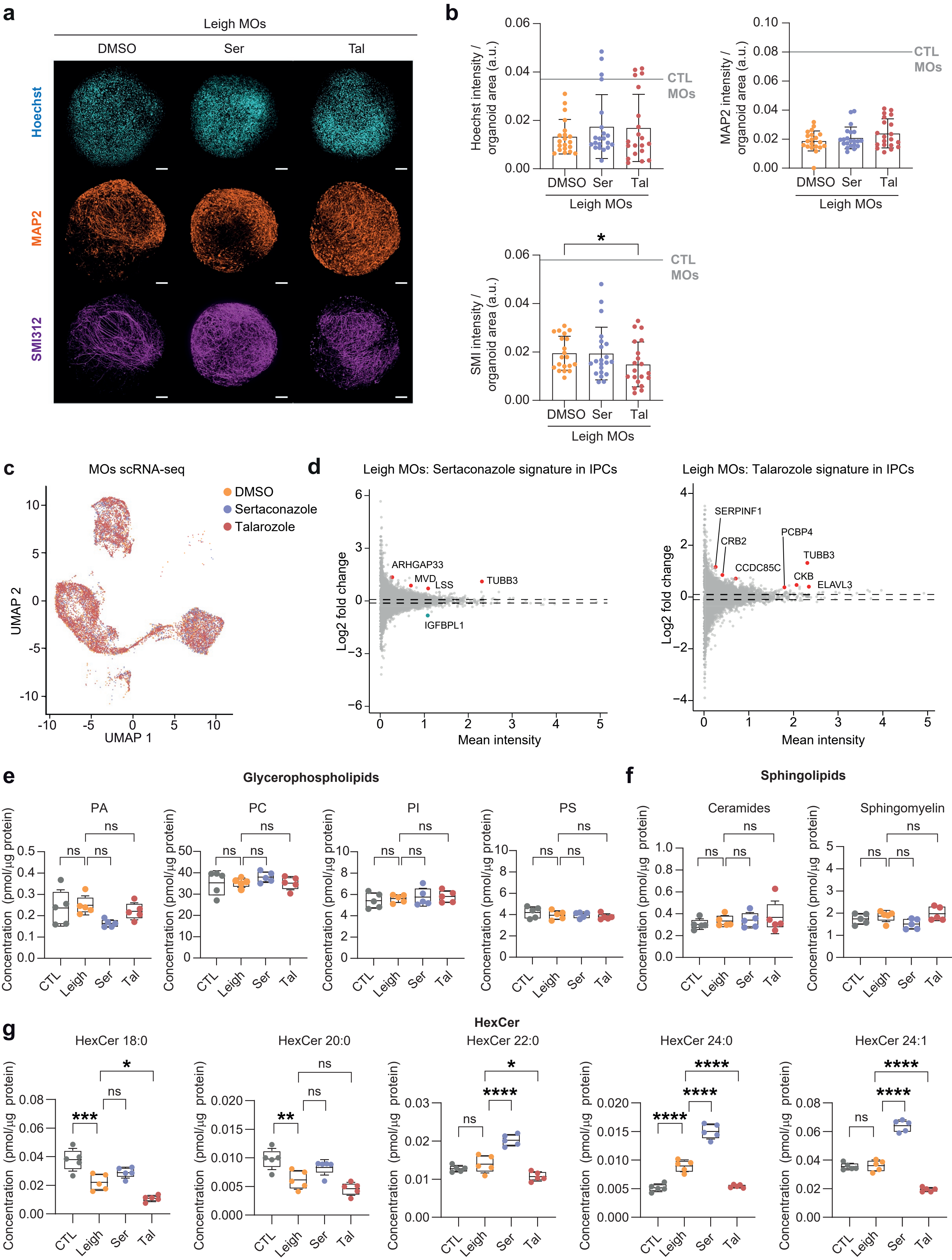

a
Leigh MOs
DMSO
Ser
Tal
Hoechst
MAP2
SMI312
b
Hoechst intensity / organoid area (a.u.)
MAP2 intensity / organoid area (a.u.)
SMI intensity / organoid area (a.u.)
CTL MOs
Leigh MOs
c
MOs scRNA-seq
DMSO
Sertaconazole
Talarozole
UMAP 1
UMAP 2
d
Leigh MOs: Sertaconazole signature in IPCs
Leigh MOs: Talarozole signature in IPCs
ARHGAP33
MVD
LSS
TUBB3
IGFBPL1
SERPINF1
CRB2
PCBP4
CCDC85C
CKB
ELAVL3
Log2 fold change
Mean intensity
e
Glycerophospholipids
PA
PC
PI
PS
f
Sphingolipids
Ceramides
Sphingomyelin
g
HexCer
HexCer 18:0
HexCer 20:0
HexCer 22:0
HexCer 24:0
HexCer 24:1
Concentration (pmol/µg protein)
CTL
Leigh
Ser
Tal
ns

Supplementary Figure 7. Sertaconazole and talarozole modulate lipid metabolism in Leigh neural models (related to Figure 4 and Figure 5). (a-b) Organization and quantification of Hoechst, MAP2 and SMI312 intensities in treated Leigh MOs. Bar plots: mean +/- SD of signal intensity normalized to organoid area. Dots: individual MOs (DMSO: 20 MOs, Ser: 21 MOs, Tal: 20 MOs); n=6 independent experiments; *p<0.05, unpaired two-tailed t test, compound-treated Leigh MOs vs DMSO-treated Leigh MOs. Scale bars: 100 µm. **(c)** UMAP plot of scRNAseq of Leigh MOs treated with DMSO, sertaconazole, or talarozole. **(d)** Volcano plots highlighting differentially expressed genes (DEG) in IPCs within Leigh MOs treated with sertaconazole (left) or talarozole (right). **(e-g)** Targeted lipidomics in CTL NPCs and Leigh NPCs treated for 24 h with DMSO, 10 µM sertaconazole, or 10 µM talarozole displaying the concentration of total species of glycerophospholipids, sphingolipids and hexosylceramides (HexCer). Boxplots: mean (center), 25/75 percentiles (box), and 1x SD (whiskers). Dots: biological replicates (n=5); ns: not significant, *p<0.05, **p<0.01, ***p<0.005; one-way ANOVA. Source data are provided as a Source Data file.

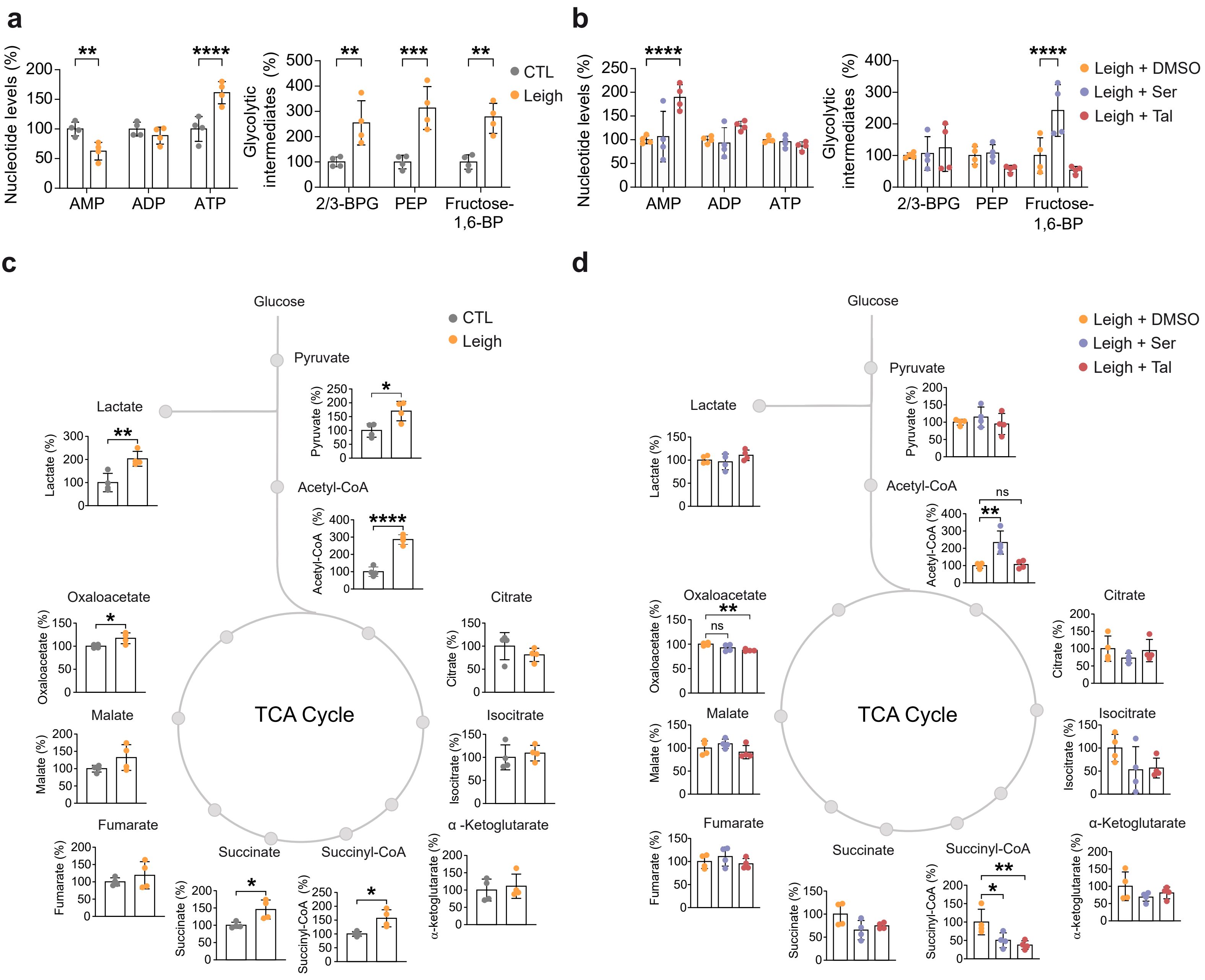

a
Nucleotide levels (%)
AMP
ADP
ATP
Glycolytic intermediates (%)
2/3-BPG
PEP
Fructose-1,6-BP
CTL
Leigh
b
Nucleotide levels (%)
AMP
ADP
ATP
Glycolytic intermediates (%)
2/3-BPG
PEP
Fructose-1,6-BP
Leigh + DMSO
Leigh + Ser
Leigh + Tal
c
Glucose
CTL
Leigh
Pyruvate
Pyruvate (%)
Lactate
Lactate (%)
Acetyl-CoA
Acetyl-CoA (%)
Oxaloacetate
Oxaloacetate (%)
Citrate
Citrate (%)
TCA Cycle
Malate
Malate (%)
Isocitrate
Isocitrate (%)
Fumarate
Fumarate (%)
α -Ketoglutarate
α-ketoglutarate (%)
Succinate
Succinate (%)
Succinyl-CoA
Succinyl-CoA (%)
d
Glucose
Leigh + DMSO
Leigh + Ser
Leigh + Tal
Pyruvate
Pyruvate (%)
Lactate
Lactate (%)
Acetyl-CoA
Acetyl-CoA (%)
ns
Oxaloacetate
Oxaloacetate (%)
ns
Citrate
Citrate (%)
TCA Cycle
Malate
Malate (%)
Isocitrate
Isocitrate (%)
Fumarate
Fumarate (%)
α-Ketoglutarate
α-ketoglutarate (%)
Succinate
Succinate (%)
Succinyl-CoA
Succinyl-CoA (%)

Supplementary Figure 8. Effect of sertaconazole and talarozole on energy metabolism in Leigh NPCs (related to Figure 5). (a-b) Targeted metabolomics for ATP and glycolysis-related metabolites in CTL NPCs vs Leigh NPCs, and in DMSO-treated Leigh NPCs vs 10 µM compound-treated Leigh NPCs for 24 h. Bar plots: mean +/- SD. Dots: biological replicates; n=4 independent experiments; ns: not significant, **p<0.01, ***p<0.005, ****p<0.001; unpaired two-tailed t test for (a) and one-way ANOVA for (b). **(c-d)** Targeted metabolomics for TCA cycle-related metabolites in control NPCs vs Leigh NPCs (left) and in Leigh NPCs treated with DMSO, 10 µM sertaconazole, or 10 µM talarozole for 24 h (right). Bar plots: mean +/- SD. Dots: biological replicates; n=4 independent experiments; ns, **p<0.01, ***p<0.005, ****p<0.001; unpaired two-tailed t test for (c) and one-way ANOVA for (d). Source data are provided as a Source Data file.

a

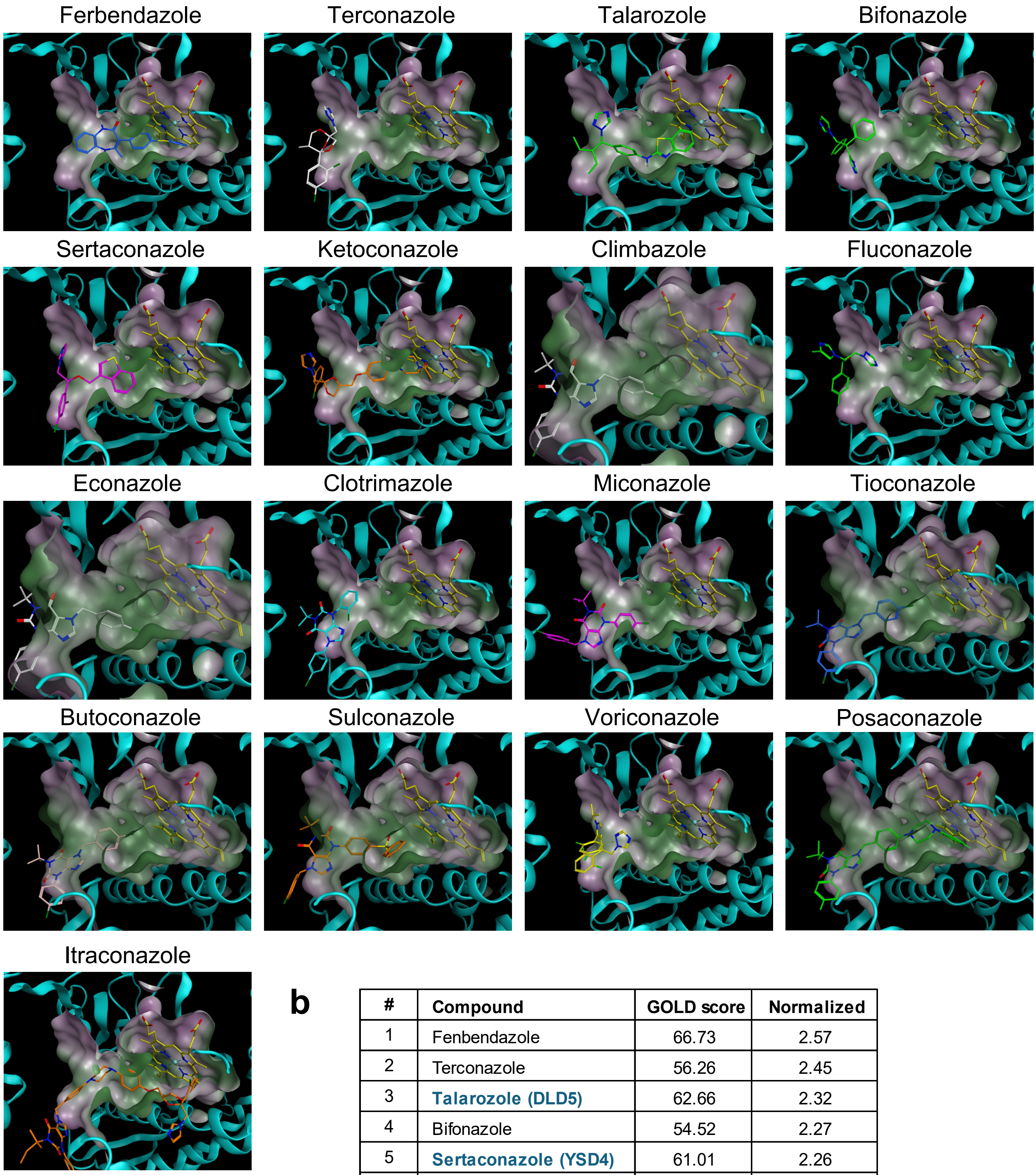

b

#	Compound	GOLD score	Normalized
1	Fenbendazole	66.73	2.57
2	Terconazole	56.26	2.45
3	**Talarozole (DLD5)**	62.66	2.32
4	Bifonazole	54.52	2.27
5	**Sertaconazole (YSD4)**	61.01	2.26
6	Ketoconazole	79.49	2.20
7	Climbazole	62.89	2.17
8	**Fluconazole (YSD2)**	45.29	2.06
9	Econazole	59.95	2.00
10	Clotrimazole	57.00	1.97
11	Miconazole	60.28	1.94
12	Tioconazole	56.87	1.90
13	Butoconazole	55.61	1.85
14	Sulconazole	69.13	1.82
15	Voriconazole	54.97	1.72
16	Posaconazole	70.37	1.64
17	**Itraconazole (YSD3)**	67.71	1.09

Supplementary Figure 9. Talarozole and sertaconazole outperform other azoles in binding CYP120 pocket (related to Figure 6). (a) Docking modeling prediction for 17 different azole compounds with the configuration fixed on the CYP120 pocket. **(b)** List of azole compounds modeled in (a) ranked based on the normalized GOLD score to the number of carbons of each azole compound showing talarozole (DLD5) and sertaconazole (YSD4) among best performing azole compounds.

a

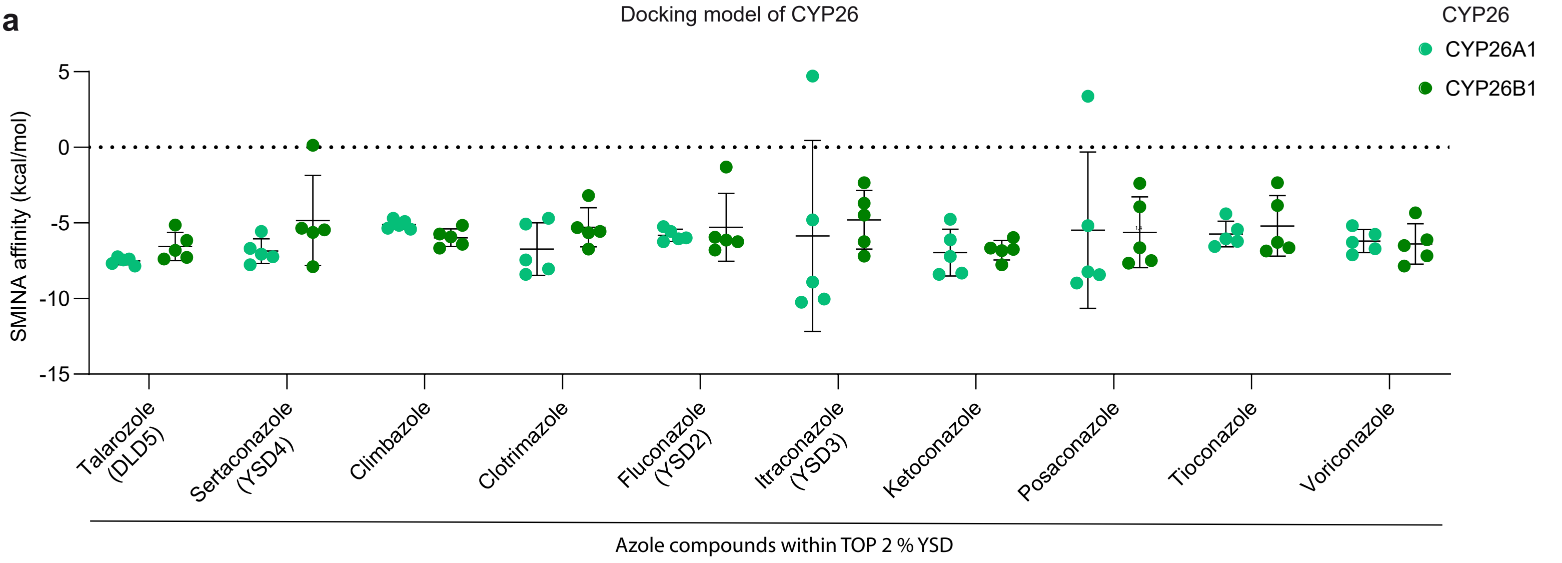

Docking model of CYP26
CYP26
CYP26A1
CYP26B1
SMINA affinity (kcal/mol)
5
0
-5
-10
-15
Talarozole (DLD5)
Sertaconazole (YSD4)
Climbazole
Clotrimazole
Fluconazole (YSD2)
Itraconazole (YSD3)
Ketoconazole
Posaconazole
Tioconazole
Voriconazole
Azole compounds within TOP 2 % YSD

b

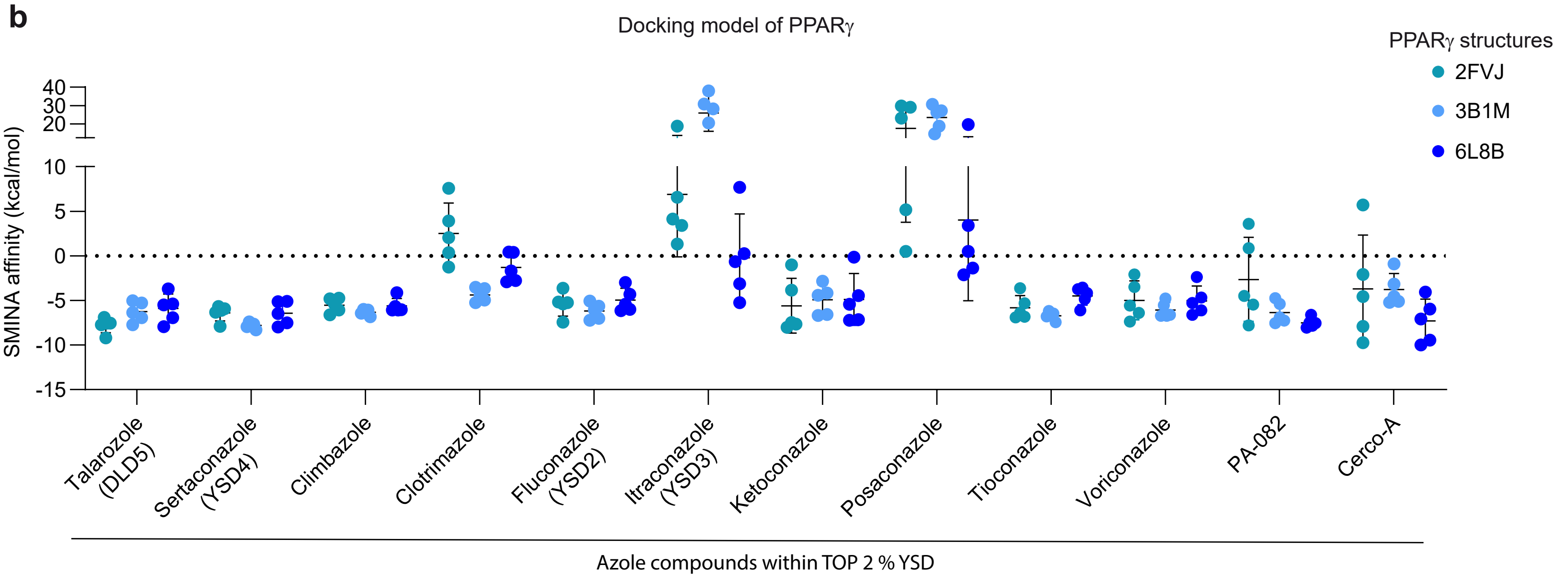

Docking model of PPARγ
PPARγ structures
2FVJ
3B1M
6L8B
SMINA affinity (kcal/mol)
40
30
20
10
5
0
-5
-10
-15
Talarozole (DLD5)
Sertaconazole (YSD4)
Climbazole
Clotrimazole
Fluconazole (YSD2)
Itraconazole (YSD3)
Ketoconazole
Posaconazole
Tioconazole
Voriconazole
PA-082
Cerco-A
Azole compounds within TOP 2 % YSD

Supplementary Figure 10. SMINA docking affinity scores of talarozole and sertaconazole to CYP26 and PPARγ compared to other azole compounds (related to Figure 6). (a-b) Docking results of 10 different azole compounds in binding to CYP26 or PPARγ proteins using SMINA affinity score. Scatter plot: mean +/- SD. Dots: top 5 Diffdock poses, re-docked, and energy-minimized with SMINA. The ligands PA-082 and Cerco-A, co-crystallized with PPARγ structures 2FVJ and 3B1M, respectively, are shown for comparison in (b). Source data are provided as a Source Data file.

a

SNPH (MW: 54 kDa) KIF5A (MW: 118-120 kDa) β-actin (MW: 42kDa)

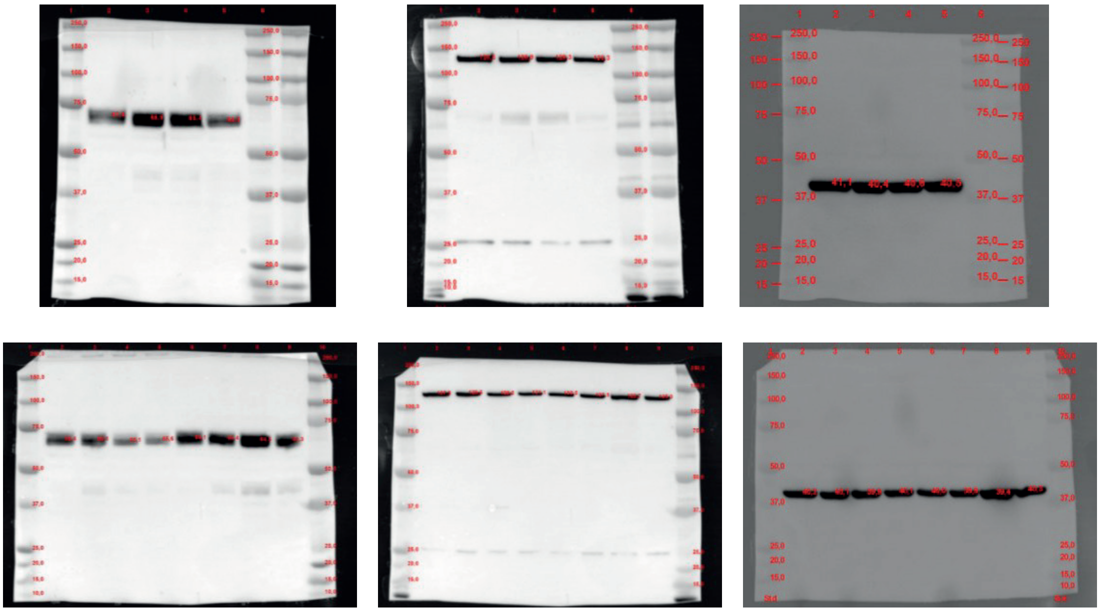

1: Marker
2: CTL DMSO
3: Leigh + DMSO
4: Leigh + Sertaconazole
5: Leigh + Talarozole
6: Marker

1: Marker
2: CTL DMSO
3: Leigh + DMSO
4: Leigh + Sertaconazole
5: Leigh + Talarozole
6: CTL DMSO
7: Leigh + DMSO
8: Leigh + Sertaconazole
9: Leigh + Talarozole
10: Marker

b

SNPH (MW: 54 kDa) KIF5A (MW: 118-120 kDa) β-actin (MW: 42kDa)

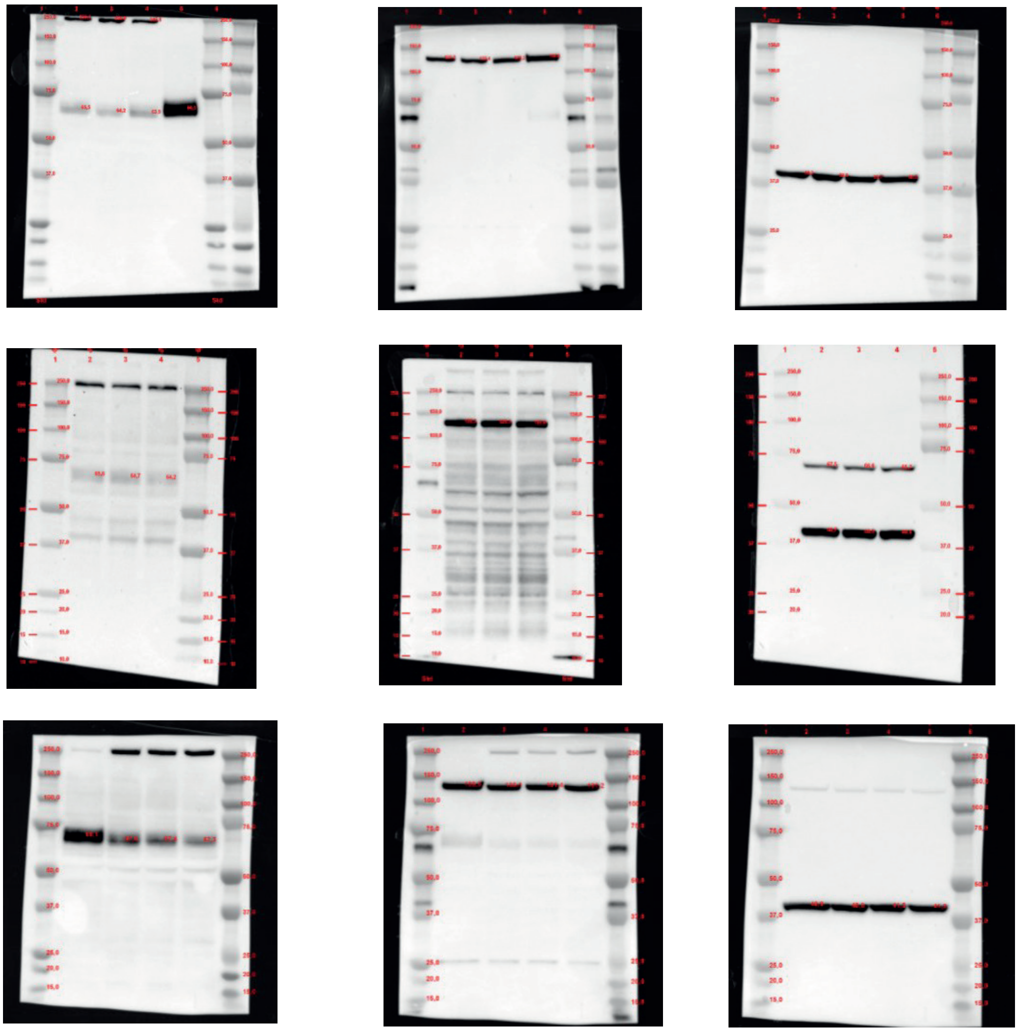

1: Marker
2: Leigh + DMSO
3: Leigh + Talarozole
4: Leigh + Sertaconazole
5: Control + DMSO
6: Marker

1: Marker
2: Leigh + DMSO
3: Leigh + Talarozole
4: Leigh + Sertaconazole
5: Marker

1: Marker
2: CTL DMSO
3: Leigh + DMSO
4: Leigh + Sertaconazole
5: Leigh + Talarozole
6: Marker

Supplementary Figure 11. Original blots for SNPH, KIF5A and β-Actin (related to Supplementary Figure 6). a) Unprocessed blot scans of treated mDANs (related to Supplementary Figure 6i) for batches 1 (top row) 2 and 3 (bottom row). **b)** Unprocessed blot scans of treated MOs (related to Supplementary Figure 6k) for batches 1 (top row), 2 (middle row) and 3 (bottom row).

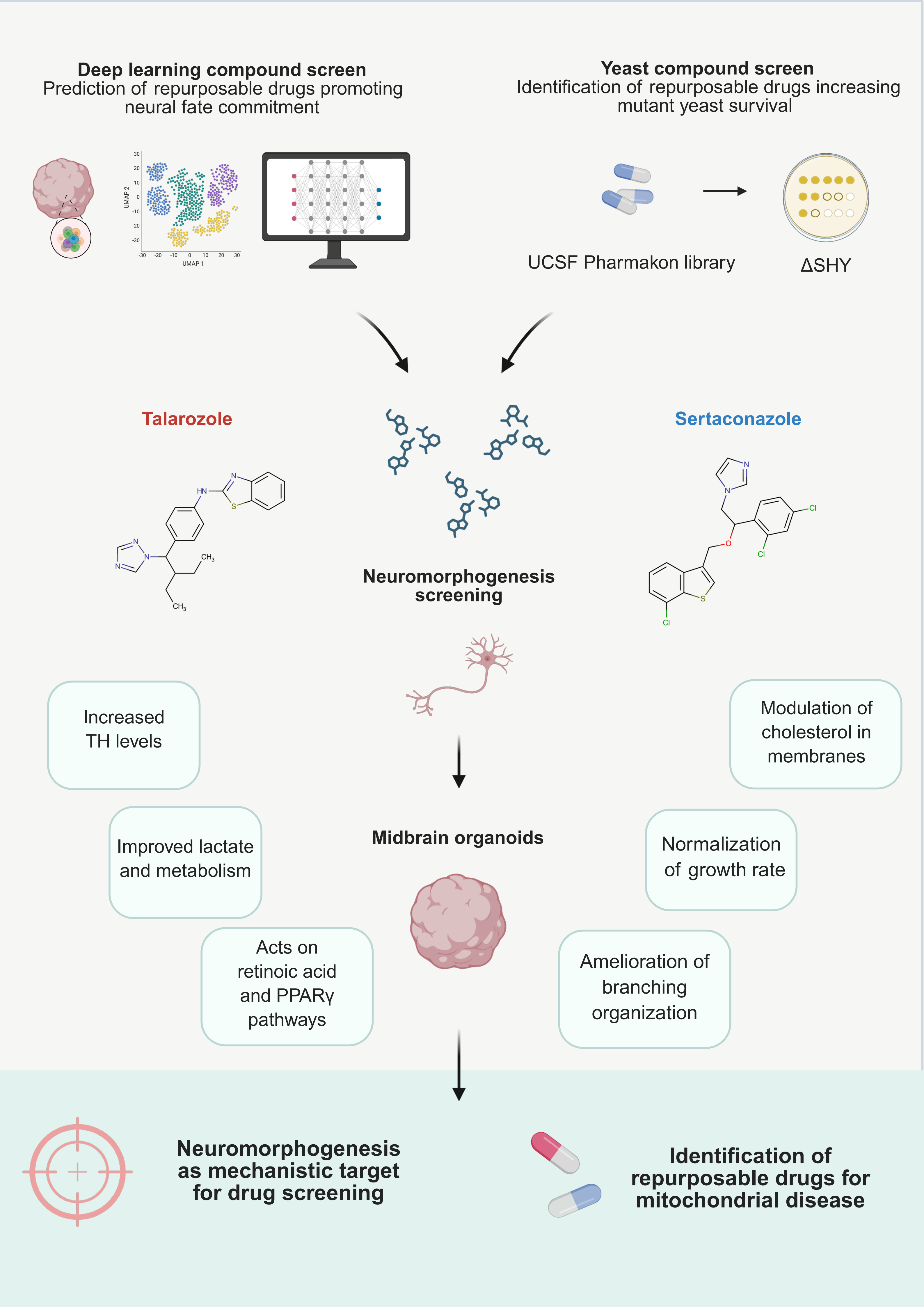

Deep learning compound screen
Prediction of repurposable drugs promoting neural fate commitment
UMAP 1
UMAP 2
Yeast compound screen
Identification of repurposable drugs increasing mutant yeast survival
UCSF Pharmakon library
ΔSHY
Talarozole
Sertaconazole
Neuromorphogenesis screening
Increased TH levels
Improved lactate and metabolism
Acts on retinoic acid and PPARγ pathways
Modulation of cholesterol in membranes
Normalization of growth rate
Amelioration of branching organization
Midbrain organoids
Neuromorphogenesis as mechanistic target for drug screening
Identification of repurposable drugs for mitochondrial disease

Supplementary Figure 12. Overview of the study. Cartoon depicting the results of this work. Using deep learning and yeast-based screens, we identified azole compounds talarozole and sertaconazole as potential repurposable drugs for Leigh based on their capacity to promote neuronal morphogenesis and ameliorate aspects related to neuronal metabolism (created in BioRender. Menacho, C. (2026) https://BioRender.com/m7lhoz8).

Supplementary Table 1. Small molecule screening data in yeast

Category	Parameter	Description
Assay	Type of assay	Cell-based phenotypic yeast growth/viability HTS assay (luminescence endpoint) in 384-well plates.
	Target	Saccharomyces cerevisiae SURF1 homolog deficiency model: SHY1 deletion (shy1Δ::KANr) in BY4743 S288C background (yeast avatar for SURF1 deficiency). Screen conditions: YP + 2% lactate, 30°C, start OD600=0.025, 24 h.
	Primary measurement	Luminescence endpoint readout of yeast growth/viability (ATP proxy) using BacTiter-Glo.
	Key reagents	Yeast strains: BY4743 WT (control) and BY4743 shy1Δ (SURF1 homolog KO). Media: YP + 2% lactate. Reagent: BacTiter-Glo (Promega). Vehicle: DMSO (0.72% v/v in vehicle controls; matched across plates).
	Assay protocol	Compounds (DMSO stocks) were acoustically transferred into 384-well assay plates at 20 µM final: 50 nL from the SMDC/Pharmakon drug collection or 200 nL from the TargetMol L4000 library using a Labcyte Echo 650. Yeast cells from overnight cultures were resuspended in YP + 2% lactate to an optimized OD600 (for SHY1: start OD600=0.025) and dispensed (25 µL; 20 µL for TargetMol) using a BioTek EL406. Plates were incubated at 30°C for 24 h (SHY1). BacTiter-Glo was dispensed (25 µL; 20 µL for TargetMol), plates briefly vortexed, incubated 10–50 min, then read on an EnVision luminescence plate reader.
	Additional comments	Prior to screening, seeding density and BacTiter-Glo kinetics were optimized; proceed only with robust assay performance (Z′>0.5).
Library	Library size	Two libraries were screened: TargetMol L4000 Bioactive Compound Library (8,387 compounds) and Pharmakon/SMDC Drug collection (2,179 compounds).
	Library composition	Repurposing-focused collections: (1) TargetMol L4000: annotated bioactive small molecules (includes approved, clinical, and preclinical subsets); (2) Pharmakon/SMDC: curated drug-like/repurposing compound collection.
	Source	TargetMol Chemicals Inc. (L4000 Bioactive Compound Library) and Pharmakon/Small Molecule Discovery Center (SMDC) Drug collection.
	Additional comments	Libraries were screened in 384-well format. Compounds were pre-spotted/acoustically transferred (Echo 650) in DMSO to achieve 20 µM final. Transfer volumes differed by library (50 nL SMDC/Pharmakon; 200 nL TargetMol).
Screen	Format	Single-point primary screen in 384-well plates using both TargetMol L4000 and Pharmakon/SMDC libraries.
	Concentration(s) tested	Primary screen: 20 µM final (single point), 0.72% DMSO (v/v) vehicle controls. Confirmatory testing: 8-point, 2-fold dose–response starting at 40 µM (40, 20, 10, 5 µM, etc.) in triplicate; DMSO control at 0.72%.
	Plate controls	Negative control (mutant baseline): shy1Δ + DMSO. Positive control (max growth reference): WT + DMSO. Controls were included on each plate (e.g., multiple wells per control).
	Reagent/ compound dispensing system	Compounds: acoustic transfer (Labcyte Echo 650). Yeast suspension and BacTiter-Glo: BioTek EL406 automated dispenser.
	Detection instrument and software	EnVision plate reader (luminescence) and associated reader software.

<table>
<tr><td></td><td>Assay validation/QC</td><td>Assay quality monitored by Z′ per plate; advance screens only after achieving Z′>0.5 during optimization and maintaining acceptable Z′ during screening.</td></tr>
<tr><td></td><td>Correction factors</td><td>Background subtraction; optional positional-bias correction if needed (e.g., B-score).</td></tr>
<tr><td></td><td>Normalization</td><td>Plate-based normalization to vehicle controls; hits scored relative to the plate distribution. Report both Z-score and %Rescue vs mutant baseline when applicable. Hit cutoff: mean ± 3σ (as per workflow).</td></tr>
<tr><td></td><td>Additional comments</td><td>Incubation for SHY1: 24 h at 30°C in YP + 2% lactate (start OD600=0.025). BacTiter-Glo incubation: 10–50 min prior to read. Primary-screen replicate scheme not specified in Methods; dose–response confirmation was performed in triplicate.</td></tr>
<tr><td>Post-HTS analysis</td><td>Hit criteria</td><td>Primary hits called using mean ± 3σ cutoff relative to the plate distribution (rescuers above mean +3σ; sensitizers below mean −3σ), after QC (Z′).</td></tr>
<tr><td></td><td>Hit rate</td><td><table><tr><td></td><td>Screen</td><td>No. compounds</td><td>No. of hits</td><td>Hit rate %</td></tr><tr><td>Haploid</td><td>SMDC library</td><td>2179</td><td>58</td><td>2.66</td></tr><tr><td>Diploid</td><td>SMDC library</td><td>2179</td><td>53</td><td>2.43</td></tr><tr><td>Diploid</td><td>TargetMol</td><td>8641</td><td>70</td><td>0.81</td></tr></table></td></tr>
<tr><td></td><td>Additional assay(s)</td><td>Confirmatory dose–response in yeast (triplicate) for selected hits</td></tr>
<tr><td></td><td>Confirmation of hit purity and structure</td><td>For dose-response hits, fresh compounds were ordered. Hit identities/purities based on vendor QC.</td></tr>
<tr><td></td><td>Additional comments</td><td></td></tr>
</table>

Additional Supplementary Data

Supplementary Data 1. Test datasets used for DL-based framework analysis.

Supplementary Data 2. Clusters identified by single-cell RNA sequencing (scRNAseq) of midbrain organoids (MOs) derived from Leigh and isogenic controls. Two-sided Wilcoxon rank-sum test; Benjamini–Hochberg FDR-adjusted P values.

Supplementary Data 3. Differentially expressed genes (DEG) for each of the clusters identified by scRNAseq in Leigh MOs compared to isogenic control MOs. Two-sided Wilcoxon rank-sum test; Benjamini–Hochberg FDR-adjusted P values.

Supplementary Data 4. Gene ontology (GO) biological processes identified by scRNAseq in Leigh MOs compared to isogenic control MOs and Leigh MOs treated with talarozole or sertaconazole. One-sided Fisher's exact test; Benjamini–Hochberg FDR-adjusted P values.

Supplementary Data 5. Lipidomics of Leigh NPCs compared to isogenic control NPCs.

Supplementary Data 6. List of reagents, antibodies, and primers used throughout the experiments.

Supplementary Data 7. List of training and validation datasets for DL-based framework analysis.
