# Supplementary material for: Accelerating Leigh syndrome drug discovery through deep learning screening in brain organoids
Source: Nat Commun. 2026 Apr 20;17:3570. doi: 10.1038/s41467-026-71391-2 (PMC13096141; doi:10.1038/s41467-026-71391-2)
Supplement: Supplementary file 2 — Description of Additional Supplementary Files [file 41467_2026_71391_MOESM2_ESM.pdf]

Description of Additional Supplementary Files

File name: Supplementary Data 1
Description: Test datasets used for DL-based framework analysis.

File name: Supplementary Data 2
Description: Clusters identified by single-cell RNA sequencing (scRNAseq) of midbrain organoids (MOs) derived from Leigh and isogenic controls. Two-sided Wilcoxon rank-sum test; Benjamini–Hochberg FDR-adjusted P values.

File name: Supplementary Data 3
Description: Differentially expressed genes (DEG) for each of the clusters identified by scRNAseq in Leigh MOs compared to isogenic control MOs. Two-sided Wilcoxon rank-sum test; Benjamini–Hochberg FDR-adjusted P values.

File name: Supplementary Data 4
Description: Gene ontology (GO) biological processes identified by scRNAseq in Leigh MOs compared to isogenic control MOs and Leigh MOs treated with talarozole or sertaconazole. One-sided Fisher's exact test; Benjamini–Hochberg FDR-adjusted P values.

File name: Supplementary Data 5
Description: Lipidomics of Leigh NPCs compared to isogenic control NPCs.

File name: Supplementary Data 6
Description: List of reagents, antibodies, and primers used throughout the experiments.

File name: Supplementary Data 7
Description: List of training and validation datasets for DL-based framework analysis.
